# Lineage‐specific plasmid acquisition and the evolution of specialized pathogens in *Bacillus thuringiensis* and the *Bacillus cereus* group

**DOI:** 10.1111/mec.14546

**Published:** 2018-04-02

**Authors:** Guillaume Méric, Leonardos Mageiros, Ben Pascoe, Dan J. Woodcock, Evangelos Mourkas, Sarah Lamble, Rory Bowden, Keith A. Jolley, Ben Raymond, Samuel K. Sheppard

**Affiliations:** ^1^ The Milner Centre for Evolution Department of Biology and Biochemistry University of Bath Bath UK; ^2^ Institute of Life Science Swansea University Medical School Swansea UK; ^3^ MRC CLIMB Consortium University of Bath Bath UK; ^4^ Mathematics Institute and Zeeman Institute for Systems Biology and Infectious Epidemiology Research University of Warwick Coventry UK; ^5^ Wellcome Trust Centre for Human Genetics University of Oxford Oxford UK; ^6^ Department of Zoology University of Oxford Oxford UK; ^7^ Department of Life Sciences Faculty of Natural Sciences Imperial College London Ascot UK; ^8^ Department of Biosciences University of Exeter Exeter UK

**Keywords:** *Bacillus cereus*, *Bacillus thuringiensis*, insecticidal toxins, mobile genetic elements, pan‐genome

## Abstract

Bacterial plasmids can vary from small selfish genetic elements to large autonomous replicons that constitute a significant proportion of total cellular DNA. By conferring novel function to the cell, plasmids may facilitate evolution but their mobility may be opposed by co‐evolutionary relationships with chromosomes or encouraged via the infectious sharing of genes encoding public goods. Here, we explore these hypotheses through large‐scale examination of the association between plasmids and chromosomal DNA in the phenotypically diverse *Bacillus cereus* group. This complex group is rich in plasmids, many of which encode essential virulence factors (Cry toxins) that are known public goods. We characterized population genomic structure, gene content and plasmid distribution to investigate the role of mobile elements in diversification. We analysed coding sequence within the core and accessory genome of 190 *B. cereus* group isolates, including 23 novel sequences and genes from 410 reference plasmid genomes. While *cry* genes were widely distributed, those with invertebrate toxicity were predominantly associated with one sequence cluster (clade 2) and phenotypically defined *Bacillus thuringiensis*. Cry toxin plasmids in clade 2 showed evidence of recent horizontal transfer and variable gene content, a pattern of plasmid segregation consistent with transfer during infectious cooperation. Nevertheless, comparison between clades suggests that co‐evolutionary interactions may drive association between plasmids and chromosomes and limit wider transfer of key virulence traits. Proliferation of successful plasmid and chromosome combinations is a feature of specialized pathogens with characteristic niches (*Bacillus anthracis*,* B. thuringiensis*) and has occurred multiple times in the *B. cereus* group.

## 1 INTRODUCTION

A recurring feature of the genome organization of many pathogenic bacteria is that important virulence factors are often encoded on horizontally mobile genetic elements (MGEs) (Hacker & Carniel, 2001; Sansonetti, Kopecko, & Formal, 1981; Smith, 2001). A simplistic argument for the location of the genes in the “accessory genome” is that the products they encode are periodically beneficial, as might be the case for opportunistic pathogens with facultative environmental niches (Eberhard, 1990). However, theory indicates that if genes are beneficial overall, then selection will favour transfer of these genes into the chromosome to avoid the costs of plasmid carriage (Bergstrom, Lipsitch, & Levin, 2000). Moreover, many pathogenic bacteria carry essential virulence genes on plasmids, sometimes even when there is ecological and genomic evidence indicating that they are obligate pathogens or compete and replicate poorly in the environment (Hugh‐Jones & Blackburn, 2009; Keim et al., 2009; Yang, 2005; Yara, Kunimi, & Iwahana, 1997).

There are several competing, although not necessarily mutually exclusive, hypotheses that explain why some genes are carried on mobile elements and why bacterial virulence factors, in particular, tend to be mobile. These include the theory that hot spots for recombination occur in the accessory genome. Nonhomologous recombination in the accessory genome may have less costly consequences for overall fitness of the cell, and there is widespread evidence of substantial recombination in the evolution of bacterial virulence genes (de Maagd, Bravo, Berry, Crickmore, & Schnepf, 2003; Lawrence, 2005). Furthermore, genes may be able to persist in plasmids through hitch‐hiking with beneficial genes or alleles ensuring that plasmids are maintained by periodically rising to high frequencies via selection on these traits (Bergstrom et al., 2000). Both the recombination and hitch‐hiking theories may be pertinent for pathogenic bacteria, which are expected to be subject to intense and ongoing selection pressure via host–parasite co‐evolution (Lawrence, 2005). Another explanation would be that plasmid genes are generally present in higher copy numbers than chromosomal genes, which may result in the persistence of fitness‐enhancing genes that would be beneficial during highly selective events. This has been demonstrated to some extent for antibiotic resistance genes carried on plasmids (Huang et al., 2013; San Millan et al., 2015).

One theory that explains the particular mobility of bacterial virulence genes is “infectious cooperation.” Many bacterial virulence factors are secreted, and costly. Secreted virulence factors can be exploited by social “cheaters” that fail to invest in virulence, and these cheaters can outcompete more virulent producers within hosts (West, Diggle, Buckling, Gardner, & Griffin, 2007). Infecting cheating bacteria with plasmids or MGEs carrying virulence genes can convert cheaters to cooperators, a process that can theoretically improve transmission and alter population structure to favour cooperative virulence (Rankin, Rocha, & Brown, 2010; Smith, 2001). Synthetic experiments (Dimitriu et al., 2014) and the recent evolutionary origin of genes for secreted products provide some support for this theory (Nogueira et al., 2009), and major classes of virulence factors can be cooperative public goods, including Cry toxins, quorum‐sensing signals and quorum‐regulated virulence factors in the *Bacillus cereus* group (Deng et al., 2015; Raymond, West, Griffin, & Bonsall, 2012; Zhou, Slamti, Nielsen‐Leroux, Lereclus, & Raymond, 2014).

The *B. cereus* group has adapted and radiated to exploit environmental niches and a taxonomically broad array of hosts to an extent that can be matched by few known pathogens (Raymond & Bonsall, 2013). Hosts for *B. cereus* sensu stricto (*Bc*)*, Bacillus thuringiensis* (*Bt*) and *Bacillus anthracis* (*Ba*) include vertebrates, insects and nematodes (Raymond & Bonsall, 2013; Raymond, Johnston, Nielsen‐Leroux, Lereclus, & Crickmore, 2010; Ruan, Crickmore, Peng, & Sun, 2015; Turnbull, 2002), while plants have been implicated as vectors of entomopathogenic strains (Raymond, Wyres, Sheppard, Ellis, & Bonsall, 2010). This adaptive radiation means that this group is of broad significance, containing strains important for insect pest management, food production and human health. This provides an opportunity for studying how ecology in diverse pathogenic niches shapes bacterial genomes, especially as a large number of *B. cereus* genotypes are associated with well‐characterized environmental and host niches (Guinebretière et al., 2008, 2010; Raymond & Bonsall, 2013; Raymond, Wyres, et al., 2010). *Bacillus* plasmids show great diversity and variety, can be large size and are thought to be involved in many processes (Zheng, Peng, Ruan, & Sun, 2013; Zheng et al., 2015, 2017). Importantly, several characteristic and essential virulence factors are encoded on plasmids in *B. cereus* sensu lato, a group which includes *B. cereus* sensu stricto (*Bc*), *Bt*,* Ba* and which is collectively referred to as the *B. cereus* group (Gonzalez, Brown, & Carlton, 1982; Okinaka et al., 1999).

Within the *B. cereus* group, the species designation *Bt* is defined by the possession of proteinaceous inclusion bodies, mainly formed of the essential virulence factors known as Cry (Crystal) toxins. These are large, pore‐forming proteins that enable orally ingested bacteria to invade the invertebrate haemolymph from the midgut (Schnepf et al., 1998). These toxins cause paralysis and are lethal at high doses, but are relatively host‐specific and have no known toxicity to vertebrates, hence their widespread incorporation into genetically modified insect‐resistant crops (Bravo, Likitvivatanavong, Gill, & Soberon, 2011). The *B. cereus* group possesses a rich diversity of accessory genome elements with numerous large conjugative plasmids (Hu, Van der Auwera, Timmery, Zhu, & Mahillon, 2009; Van der Auwera & Mahillon, 2008; Zheng et al., 2013). *Bacillus cereus* group isolates can contain a large number of plasmids, and this plasmid complement can vary substantially both within and between serotypes (Hu, Swiecicka, Timmery, & Mahillon, 2009; Reyes‐Ramirez & Ibarra, 2008), indicating that the accessory genome has the potential to respond rapidly to ecological change.

Defining a species based on the possession of horizontally mobile *cry* genes is problematic. Unsurprisingly, *Bt* is not a monophyletic group, and several divergent clades defined by multilocus sequence typing (MLST) or genomic data contain *Bt* isolates with Cry inclusions (Raymond, Wyres, et al., 2010). The taxonomy of the *B. cereus* group*,* and of *Bt* within it, is controversial, while accurate and informative species delineation has important economic implications (EFSA 2016, Raymond & Federici, 2017). The licensing and “safe” status of *Bt* as a biological control agent that can be applied to vegetable crops are partly dependent on its biological distinctiveness from human pathogenic *Bc* and *Ba*. Although *Bt*‐based products are considered to be among the safest insecticides on the market (Federici & Siegel, 2007; Siegel, 2001), this reputation can be damaged by uncertain taxonomy and lack of rigour in interpreting epidemiological evidence (Raymond & Federici, 2017). Moreover, the possible horizontal mobility of virulence factors from vertebrate pathogens within the *B. cereus* group to invertebrate pest control agents also has potential safety implications for the use of *Bt* in biocontrol (EFSA 2016).

The aims of this study were threefold. First, to use a revised pan‐genomic analysis to assess the phylogenetic status of the *B. cereus* group. Second, to explore the mobility of key virulence gene and virulence plasmids across the group. Third, to assess whether patterns of plasmid/chromosome association in this group are consistent with current evolutionary ecology theory for plasmids and plasmid gene content.

## 2 METHODS

### 2.1 Isolate sampling and plasmid extraction

*Bt* isolates with diverse host toxicity were chosen for whole‐genome sequencing and plasmid purification. These included isolates available from the *Bacillus* Genetic Stock Centre (BGSC), the Agricultural Research Service (NRRL) culture collection, supplemented with isolates sampled for this study. Prior to sequencing, the identity of isolates with Cry inclusions was confirmed by light microscopy of sporulated cultures and cross‐checked by Sanger sequencing of flagellin genes (*Bthag*,* fliC*) using primers and conditions described in Xu and Cote (2006) and BLAST (Altschul, Gish, Miller, Myers, & Lipman, 1990) searches of at least 500 bp of both genes against the *nr* database from NCBI. One isolate, *Bt* serovar *brasiliensis* BGSC 4AY1, was excluded because production of Cry inclusions could not be confirmed. Plasmid extractions used High Speed MIDI kits (Qiagen) with 200 ml of bacterial culture and subsequent digestion with plasmid‐safe ATP‐dependent exonuclease (Epicentre) to remove linear DNA fragments, both as per manufacturer's directions.

### 2.2 Genome sequencing

A total of 190 *Bacillus* group genomes were used, including 23 *Bt* isolates that were sequenced as part of this study (Table S1). Plasmid and chromosomal DNA were extracted using the QIAamp DNA Mini Kit (QIAGEN, Crawley, UK), using manufacturer's instructions. DNA was quantified using the Quant‐iT DNA Assay Kit (Life Technologies, Paisley, UK) and a NanoDrop spectrophotometer before sequencing using an Illumina HiSeq 2500 analyser (Illumina, San Diego, CA, USA). 100‐bp short read paired‐end data were assembled using the velvet version 1.2.08 de novo assembly algorithm (Zerbino & Birney, 2008), incorporating the velvetoptimiser protocol (version 2.2.4) (https://github.com/tseemann/VelvetOptimiser) for all odd k‐mer values from 21 to 99. Scaffolding was disabled, and the minimum output contiguous sequence assembly setting was 200 bp. The average number of contiguous sequences for the 23 isolates and 10 plasmid extractions sequenced from this study was 407 ± 225 and 85 ± 32, respectively. The average assembly sequence length was 6,174,353 ± 367,182 bp for isolate whole genomes and 334,681 ± 194,229 bp for plasmid extractions (Table S2). This is consistent with published estimates of the genome size of members of the *Bacillus cereus* group. Other assembly quality metrics such as N50 were 119,242 ± 100,448 bp for isolate whole genomes and 92,176 ± 161,270 bp for plasmids. Isolates sequenced in this study were augmented with 182 genomes from public databases (available in April 2013), including reference genomes from *Bt* strain YBT020 (Zhu et al., 2011), *Bc* strain ATCC 14579 (Ivanova et al., 2003) and *Ba* strain Ames (Read et al., 2003) to give a total of 190 isolate genomes. Metadata for published isolate genomes was variable and sometimes lacked detailed sampling information, but these genomes were included to provide as much information as possible on the genomic diversity within the *Bacillus* group (Table S1). Functional predictions were made using the WebMGA COG server using rpsblast 2.2.15 on the NCBI COG database (http://weizhong-lab.ucsd.edu/metagenomic-analysis/server/cog/). Sequence type (ST) assignment from assembled genome sequences was performed using the mlst software (https://github.com/tseemann/mlst).

### 2.3 Creation of a reference pan‐genome from bacterial genomes

As in recent publications on other species (Meric, Hitchings, Pascoe, & Sheppard, 2016; Monteil et al., 2016; Morley et al., 2015; Murray et al., 2017; Yahara et al., 2017), a reference pan‐genome approach was used with gene‐by‐gene alignment, consistent with whole‐genome MLST (Jolley & Maiden, 2010; Maiden et al., 2013; Meric et al., 2014; Sheppard, Jolley, & Maiden, 2012), implemented in BIGSdb open‐source software. Briefly, the reference pan‐genome was constructed by combining the genomes of several reference strains (*Bt* strain YBT020 (Zhu et al., 2011), *Bc* strain ATCC 14579 (Ivanova et al., 2003) and *Ba* strain Ames (Read et al., 2003)) with whole‐genome annotations from all the other genomes of this study to derive a single gene list. To achieve this, all assembled genomes were submitted to the online automatic annotation pipeline RAST (Aziz et al., 2008). Rapid annotations of bacterial genomes provided by RAST rely on the curated database system SEED, in which novel annotations are provided directly by the annotations from the RAST user community (Overbeek et al., 2014). Allelic variants of unique genes were identified as duplicates, found in more than one isolate, and were removed to create the reference pan‐genome of the whole data set. Gene homology was defined using BLAST, with those found to have >70% nucleotide identity over >10% of the sequence length, considered to be homologous. This conservative sequence length threshold to distinguish genes from their allelic variants was deliberately set lower than the threshold of >50% sequence length commonly used to identify gene presence/absence, false negatives being considered less problematic than false positives in terms of characterizing pan‐genomes. Indeed, from a purely quantitative perspective, overestimating the size of the core genome is potentially equally as bad as underestimating it. However, from a methodological point of view, when defining the pan‐genome, the rigorous elimination of duplicates reduces the number of potential BLAST gene mismatches for each draft genome that is compared to the reference pan‐genome list. In real terms, this leads to more accurate quantification of the total genome size based on coding sequences. Furthermore, overestimating alleles by considering them as distinct genes is particularly problematic for downstream analyses where putative gene function is investigated. For example, bias could be introduced into broad analyses of COG/KEGG functional groups, and more detailed analyses of individual gene function would be confounded by an inflated number of paralogs. The resulting *B. cereus* reference pan‐genome was based upon all genomes listed in Table S1, which included isolates from *Bc*,* Bt* and *Ba* within the *B. cereus* group. The total number of unique genes in the pan‐genome from all these isolates was 27,016.

### 2.4 Creation of a reference plasmid gene list from 410 reference plasmid sequences

Discriminating plasmid genes from chromosomal genes is challenging using the data from high‐throughput short read sequencing that typically use total genomic DNA as a sample. This is because the reads are assembled and therefore do not produce a single read for each amplicon. To account for this, we conducted purification and separation of chromosomal and plasmid DNA prior to sequencing for 10 isolates, resulting in the sequencing of 10 plasmid sequences (Table S3). Additionally, diversity and possible genomic rearrangements among plasmids, or even their possible chromosomal integration, make gene prediction difficult without informed comparative approach to a curated reference database of known plasmid genes.

We assembled a collection from 410 full plasmid genomes, most of which were plasmids available from NCBI in September 2016 (Table S3), and were assigned as having been isolated from one of the three “species” of the *B. cereus* group based on the presumptive typed identity of the corresponding host bacteria (Table S3). Briefly, the collection comprised 81 plasmids attributed to *Bc*, 249 to *Bt* and 87 to *Ba* (consisting only of variants of pXO1 and pXO2) (Table S3). All automatically annotated genes were assembled in a single reference gene list, without any filtering of allelic variants. Indeed, mega‐plasmids, consisting of assemblages of various otherwise described plasmids, have been described in the *B. cereus* group (Zheng et al., 2013). Therefore, all genes from all plasmids were kept in the reference list to investigate whether the sequence of certain plasmids was distributed differentially among the isolates. By not filtering for allelic variants, we did not create a plasmid pan‐genome list of unique genes but maintained the plasmid sequence integrity for each plasmid, making observation of rearrangements possible, as well as being able to assess the prevalence of particular plasmid genes in given isolates. The plasmid gene list comprised 48,768 genes, some of which represented allelic variants of the same gene, for example, origins of replications, conjugation proteins and other members of the “core” plasmid genomes.

### 2.5 Core and accessory genome variation, and predicted insecticidal toxin detection

All isolate genomes were compared to the reference pan‐genome list with a locus match defined with the BLAST parameters for a positive match being >70% nucleotide identity over >50% of the sequence length (Jolley & Maiden, 2010; Meric et al., 2014; Sheppard et al., 2012). This whole‐genome MLST approach produced a matrix of gene presence/absence with different allele numbers assigned to all genes based upon nucleotide sequence, as previously described (Meric et al., 2014, 2015). The prevalence of plasmid genes, inferred from an assembled list of all genes present in 410 plasmids from NCBI, in 190 bacterial genomes was determined using BLAST as above.

Genes encoding *Bt* toxins (Cry, Cyt, Vip and Sip) were predicted via BtToxin_scanner, a tool designed to identify new candidate toxin genes from sequence data using three different kinds of prediction methods (Ye et al., 2012). This approach identified sets of candidate toxin genes in a complementary approach to the RAST/SEED pipeline presented above. Briefly, BtToxin_scanner specifically addresses challenges set by the detection of *Bt* toxins by combining a BLAST approach with an additional hidden Markov model and support vector machine approaches to accurately predict the presence of toxin genes and annotate them (Ye et al., 2012). While the RAST/SEED approach is well suited for bacterial whole genomes, care was taken for *Bt* toxins due to specific challenges such as repeats and low homology between members of the toxin families (Ye et al., 2012). These are addressed by BtToxin_scanner that specifically predicts and annotates *Bt* toxins, either as previously known variants or as novel candidate unknown toxins (Ye et al., 2012). To examine candidate genes as potential novel toxins or false positives, we proceeded as follows: low‐homology BtToxin_scanner hits of <45% amino acid sequence identity were considered good candidates, as previously described (Noguera & Ibarra, 2010), and were used as queries in protein–protein BLAST against the database of nonredundant proteins (nr) on NCBI on the 27/06/2017. When a hit in the first 50 was found to have a match with an entry annotated as Cry, or more generally any reference to predicted insecticidal activity, the hit was considered a good candidate insecticidal toxin. When no obvious predicted insecticidal‐related annotated hit was found, the protein was considered a false positive (Table S4).

### 2.6 Allelic diversity calculations

To avoid sampling bias, the number of unique alleles per isolate was calculated for randomly selected isolates. Briefly, for comparisons involving *Ba* (Figure 3a,b), for which only 17 isolates are included in this study, the number of unique alleles was determined for 17 randomly selected Cry‐positive isolates and 17 randomly selected Cry‐negative isolates. This step was repeated 50 times, and the 50 values for each group were averaged to give the final value of unique alleles per isolate in the two groups. For comparisons not involving *Ba* (Figure 3c,d,e), the number of unique alleles was determined for 50 randomly selected Cry‐positive isolates and 50 randomly selected Cry‐negative isolates. This step was repeated 50 times, and the 50 values for each group were averaged to give the final value of unique alleles per isolate in the two groups.

### 2.7 Phylogenetic and clustering analyses

Phylogenetic trees were constructed based on 2,274 core genes shared by all genomes in our data set, which were individually aligned using MAFFT (Katoh & Standley, 2013) and concatenated to produce contiguous sequence alignments in BIGSdb (Jolley & Maiden, 2010). raxml (Stamatakis, 2014) was used to reconstruct phylogenies using default parameters. Clustering of plasmid prevalence profiles was performed using the web‐based platform WebGimm (Joshi, Freudenberg, Hu, & Medvedovic, 2011) using the context‐specific infinite mixture model (Freudenberg, Sivaganesan, Wagner, & Medvedovic, 2010).

## 3 RESULTS

### 3.1 Phylogeny of *B. cereus* group isolates

To examine the phylogenetic relationships between isolates from our data set, we attributed STs to each genome using the *Bacillus cereus* MLST scheme on pubmlst (https://pubmlst.org/bcereus/) and re‐created a phylogenetic tree using raxml (Stamatakis, 2014). STs could not be assigned for five (2.6%) isolates because MLST loci were incomplete or truncated in the draft genomes. There was considerable diversity among the typable isolates with a total of 127 STs including 23 newly identified among isolates in this study. A total of 59 different STs were found in Cry‐positive and candidate Cry toxin‐harbouring isolates and 74 different STs in Cry‐negative isolates (Tables S1 and 1). Only two different STs (ST‐1 and ST‐3) were detected in the *Ba* lineage (Tables S1 and 1), which is consistent with the reported clonal nature of the population (Van Ert et al., 2007). Interestingly, eight STs (ST‐8, ST‐56, ST‐111, ST‐223, ST‐257, ST‐506, ST‐783 and ST‐934) were shared by Cry‐positive and Cry‐negative isolates, highlighting the acquisition of mobile virulence factors in divergent genetic backgrounds (Table S1). A total of 14 genomes from our data set, all initially classified as *Bc*, clustered in a clade 3 lineage with *Bacillus mycoides* and *Bacillus weihenstephanensis* isolates (Table S1, Figure S1). Two of these isolates were Cry‐positive, seven were predicted to harbour candidate novel Cry toxins and no toxin gene could be detected in five genomes (Table S1).

**Table 1:** Toxin‐harbouring and plasmid detection in different groups of isolates used in this study

Group of isolates	Number of known STs detected	Number of toxin‐positive (Cry/Vip) isolates	Number of *Ba*‐isolated plasmids detected from reference list (*n* = 87)^b^	Number of *Bc*‐isolated plasmids detected from reference list (*n* = 81)^b^	Number of *Bt*‐isolated plasmids detected from reference list (*n* = 249)^b^	Number of predicted toxin‐harbouring plasmids (Cry/Vip) detected from reference list (*n* = 66)^b^
Total data set (*n* = 190)	77	84^a^	87	28	155	33
Clade 1 (*n* = 57)	23	8	87	27	21	3
Clade 1 *Ba* only (*n* = 17)	2	0	87	3	0	0
Clade 2 (*n* = 71)	28	49	0	8	148	30
Clade 3 (*n* = 33)	10	18	0	5	10	0
Clade 4 (*n* = 17)	8	7	0	5	0	0
Clade 5 (*n* = 9)	2	2	0	5	0	0
Singletons (*n* = 3)	2	1	0	0	0	0
Cry‐positive isolates^a^ (*n* = 84)	37	84	0	15	58	33
Non‐*Ba* Cry‐negative isolates (*n* = 89)	45	0	40	17	151	7

a Does not include *Bc* strain VD022 that has been predicted to be Cry‐negative but Vip‐positive.

b The presence of a given reference plasmid was determined when >90% of the reference annotated genes were detected in a bacterial genome (containing chromosome and plasmid DNA).

**Figure 1:**
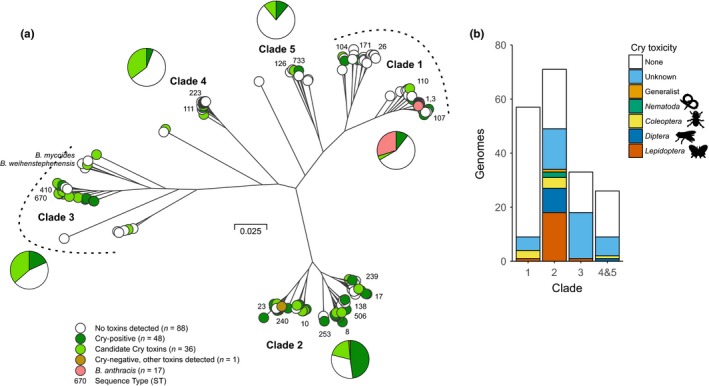
Phylogeny of 190 genomes and *cry* toxicity in the *Bacillus cereus* species complex. (a) The phylogenetic tree was reconstructed using gene‐by‐gene concatenated alignments of 2,274 core genes, and an approximation of the maximum‐likelihood algorithm implemented in RAxML. The scale represents the number of substitutions per site. Clades previously defined by multilocus sequence typing are specified in bold. *cry* endotoxin genes were identified in the genomes with BtToxin_scanner software and are indicated as present (green) or absent (white) for each genome. Isolates from the *Bacillus anthracis* clade are shown in pink. Numbers next to the tip of branches on the tree indicate sequence types from the *B. cereus* pubMLST database (https://pubmlst.org/bcereus/). (b) Inferred invertebrate host range of *B. cereus* group isolates based on known toxicity spectra of c*ry* genes present in genomes. Host range allocations are detailed in Table S1 and based on data in van Frankenhuyzen (2009) and sources within the Cry nomenclature database (Crickmore et al., 2016)) [Colour figure can be viewed at http://www.wileyonlinelibrary.com/]

A phylogenetic tree was generated from the concatenation of gene‐by‐gene alignments (Sheppard et al., 2012) of 2,274 core genes found to be shared in all genomes (Figure 1a). Most isolates clustered in clade 2 (71/190; 37.4%), which also had the highest prevalence of Cry‐positive and candidate Cry‐harbouring isolates (48/71; 67.6%) (Figure 1a, Table S1). Clades 3 and 4 had comparable prevalence of Cry‐positive and candidate Cry‐harbouring isolates (18/33; 54.5% and 7/17; 41.2%, respectively) while clade 1, comprising *Ba* isolates, had only eight of 57 (14.0%) Cry‐positive and candidate Cry‐harbouring isolates (Figure 1a, Table S1). Three isolates were not clustered in any of the MLST‐defined clades, with isolate *Bc* R309803 (ST‐74) being a singleton, and Cry‐harbouring *Bc* BAG2X1‐1 (ST‐723) and Cry‐negative *Bc* BAG2X1‐3 clustered together between clades 3 and 4 (Figure 1a).

### 3.2 Detection of candidate novel insecticidal toxins in 190 *B. cereus* group genomes

We used the BtToxin_scanner software (Ye et al., 2012) to detect the presence of genes encoding the δ‐endotoxins Cry and Cyt, and genes encoding the secreted toxins Vip and Sip in the whole‐genome sequences of 190 *Bc*,* Bt* and *Ba* isolates, including 23 new, phenotypically confirmed, *Bt* isolates. In total, the data set comprised 135 isolates identified as *Bc*, 38 as *Bt* and 17 from the *Ba* lineage. Apart from *Ba*, the species nomenclature was mostly inferred from records in the genome public repository and may include strains that are mistyped, notably for the genomes labelled as *Bc*. Predicted insecticidal toxins and predicted novel candidate Cry toxins were distributed differentially across these species designations and among previously defined clades (Raymond, Wyres, et al., 2010), (Table S1, Figure 1a). Cry, Cyt and Vip toxin genes, as well as uncharacterized candidate Cry toxins, were detected in 84 of 190 (44.2%) isolates from our data set (Table 1), including 36 of 38 (94.7%) classified as *Bt*, in 48 of 135 (35.5%) *Bc* but never in *Ba*. Notably, only 12 of 135 (8.8%) *Bc* isolates harboured previously known toxin genes, while 36 of 135 (26.6%) harboured only uncharacterized candidate toxin genes (Table S1). The fact that two strains of *Bt* (*Bt subsp. pondicheriensis* BGSC4BA1 and *Bt subsp. malaysiensis* NRRL_B23152) seemed to harbour no toxin could be due to a misclassification, but also to incomplete genomes or the presence of new toxin variants possibly not detected by our protocol. The most common Cry/Vip protein variants were Cry1Ia2, Cry2Aa9, Cry2Ab3 and Vip3A detected in six isolates, each time a combination of *Bt* and *Bc* (Table S4). Parasporins (Cry toxins with activity against cancer cells but not invertebrates) from a range of classes were detected in 10 of 190 genomes (Table S1), while many candidate Cry proteins had parasporins as the closest match (Table S4). Notably, while candidate *Cry* proteins were widely distributed across the group, those with clear invertebrate toxicity, especially to *Diptera* and *Lepidoptera,* were concentrated in clade 2 (Figure 1b). Moreover, the host taxon targeted by the Cry toxin complement in all isolates was readily identified (we identified a single generalist genome) and was typically associated with either an insect Order or nematodes, consistent with specialization on a group of hosts (Figure 1b; Table S1).

In 35 of 50 (70%) Cry/Vip‐positive isolates harbouring known characterized variants, several distinct toxin genes were detected in the same genome by BtToxin_scanner (Tables S1 and S4). This was most common in *Bt* isolates, with an average of around five (4.71 ± 3.7; *n* = 35) toxins detected per toxin‐positive genome, with four isolates predicted to harbour more than 10 detected variants (*Bt subsp. morrisoni* strain BGSC_4K1, sequenced as part of this study, had a maximum of 14 detected toxin variants in its genome). *Bc* isolates also putatively harboured several toxins, with between 2 and 3 in average per toxin‐positive genome (2.46 ± 1.80; *n* = 15), and four strains with five detected variants. In contrast, 15 of 50 (30%) isolates in total seem to only harbour one known characterized toxin variant in their genomes. For the remaining analyses of this study, we considered *B. cereus* group isolates to have a possible insecticidal activity based on the detection of known or candidate toxins rather than their assigned species in genome databases.

### 3.3 Pan‐genome variation and diversity across *B. cereus* group isolates

**Figure 2:**
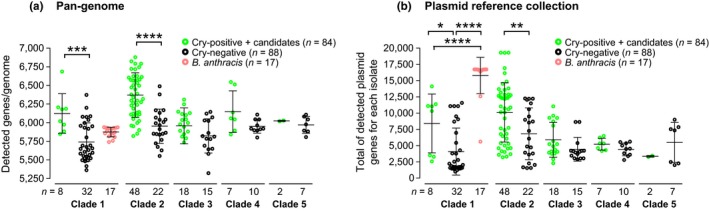
Detection of chromosomal and plasmid genes in *Bacillus cereus* group isolates. (a) Number of detected genes from a pan‐genome reference list of 27,016 genes in 190 *B. cereus* group clades and Cry‐positive and Cry‐negative groups (as defined in Figure 1). (b) Total number of detected genes from an unfiltered list of genes present in 410 full plasmids. The number of isolates within each group is indicated below each distribution plot. Significance of the difference in distribution averages was calculated after a one‐way ANOVA with Sidak's multiple comparison tests, with significance summarized as follows: ****: *p *<* *.0001, **: *p *<* *.01 [Colour figure can be viewed at http://www.wileyonlinelibrary.com/]

We then performed a complete data set‐wide pan‐genome analysis in which the presence and variation of every automatically annotated gene from every genome were examined. Gene prevalence differences were compared between various groups of isolates to examine core and accessory gene variation. An average of 6,018 (±339) genes was detected from 190 *B. cereus* group genomes from our data set. A total of 2,274 core genes were found to be present in all genomes, which represents 37.8% of the average number of genes in a *B. cereus* genome. Interestingly, the average amount of genes detected in Cry‐positive, including Cry‐candidate harbouring, isolates was always observed to be larger than in Cry‐negative isolates (Figure 2a), and this difference was significant in clade 1 (1‐way ANOVA with Sidak's multiple comparison tests, *t *=* *3.998, *df* = 175; adjusted *p *=* *.0005) and clade 2 (*t *=* *6.710, *df* = 175; adjusted *p *<* *.0001). One explanation for this observation could be the generally higher prevalence of large plasmids in Cry‐positive isolates.

Quantitative analysis of the prevalence of genes revealed that no genes were shared specifically by all Cry‐positive or by all Cry‐negative isolates. Cry toxins are a family of proteins rather than isoform variants of the same protein encoded by the same genes/alleles. This may explain why no genes were shared by all isolates. A total of 6,225 genes were found only in Cry‐positive (but not shared by all isolates) and not in Cry‐negative isolates. However, 6,172 of these were found at very low prevalence (*n* < 10 isolates), which left 53 genes present in >10 isolates (Table S5).

The dearth of genes shared at high prevalence between Cry‐positive isolates from all clades is related to the polyphyletic distribution of *cry* genes. This may be indicative of both the diversity of structure and gene content among MGEs conferring insecticidal virulence in *B. cereus* group isolates (see below) and clade‐specific insecticidal virulence associated with specific virulence factors. Nevertheless, some genes had increased prevalence in one group or the other, but this was predominantly caused by the fact that clade 2 is more significantly enriched for cry‐positive isolates than any other clade. When clade‐specific genes were examined, we found that only 21 “clade‐specific core genes” that were shared by all isolates from specific clades but absent from any other clade (1 in clade 2, 8 in clade 3 and 12 in clade 4) (Table S6). Genes specific and shared by every isolate from clade 4 notably encoded a choline‐binding protein A (CpbA), which has been shown to be an adhesion factor in Firmicutes such as *Streptococcus pneumoniae* (Luo et al., 2005) and which has been used for vaccine development (Bologa et al., 2012) (Table S6). Genes specific and shared by every isolate from clade 3 included genes encoding an uncharacterized transport system as well as genes involved in sporulation and respiration (Table S6). Notably, one gene (BC4305, annotated as hypothetical protein) was shared by all 71 isolates from clade 2 and absent from all other clades (Table S6). This gene is not located in any predicted operon in the *Bc* ATCC14579 genome, nor is it flanked by genes of known function. A total of 30 genes were found to be shared by all *Ba* and absent in the rest of the data set, including in non‐*anthracis* clade 1 isolates (Table S6), while no genes were found to be present in all non‐*anthracis* clade 1 isolates but absent in *Ba* isolates, confirming previous analyses which indicated that there are few large‐scale genomic variations that differentiate *Ba* from closely related *Bc* (Zwick et al., 2012). It is interesting to note that 50% of the pan‐genome (13,501 genes) comprised low‐frequency genes that were each present in less than four isolates, which highlights the variability of the *B. cereus* genome and is potentially related to horizontal gene transfer (HGT) in this species.

The comparison of functional prediction prevalence for different groups of genes showed that the distribution of functional categories of accessory and plasmid‐borne genes was generally similar and differed from the core genome (Figure S4). More specifically, accessory and plasmid genes were significantly enriched in prevalence from COG class L (Replication, recombination and repair) than in the core genome (Tukey's multiple comparison tests after a two‐way ANOVA; adjusted *p* = .0062 and *p* = .0021, respectively). Generally speaking, although not significantly different using a stringent statistical test, there were much lower proportions of metabolism‐associated genes (COG classes E, P and C) in accessory and plasmid genes than in the core genome (Figure S4).

### 3.4 Lower core genome allelic diversity among Cry‐positive isolates

**Figure 3:**
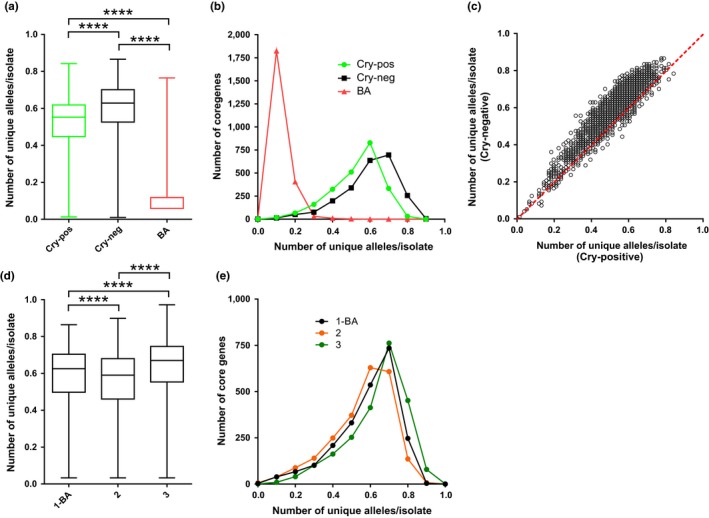
Allelic diversity of Cry‐positive and Cry‐negative *Bacillus cereus* and *Bacillus anthracis* isolates. Allelic diversity was compared by calculating the number of unique alleles per isolate for 2,192 core genes shared by all isolates of the data set. (a) Overall distribution shown as boxplots (min. to max.), with statistical significance between the distribution inferred using a Kruskal–Wallis test with Dunn's multiple comparison tests, with significances summarized as follows: ****, *p *<* *.0001. (b) Frequency distribution of core allelic diversity in each group. (c) Gene‐by‐gene comparison of allelic diversity/isolate between Cry‐positive and Cry‐negative isolates for each of 2,192 core genes (circles). The proportionality line of equal allelic diversity between the two groups is shown in red. (d) Distribution shown as boxplots (min. to max.) for each clade (1 to 3, excluding *B. anthracis* from clade 1 isolates). Each group was statistically different from one another (Kruskal–Wallis test with Dunn's multiple comparison tests; *p *<* *.0001; except clade 2 vs. clade 4 which were not; *p *=* *.1306). (d) Frequency distribution of core allelic diversity in each clade [Colour figure can be viewed at http://www.wileyonlinelibrary.com/]

We examined the allelic diversity of various groups of isolates by calculating the number of unique alleles per isolate for Cry‐positive (including Cry‐candidate harbouring), Cry‐negative and *Ba* isolates (Figure 3). We observed that these three groups had distinct distributions of allelic diversity in their core genomes (Kruskal–Wallis test with Dunn's multiple comparison tests; adjusted *p* < .0001 for each pairwise comparison of rank differences) (Figure 3a). *Ba* had much lower diversity, as expected from its clonal structure within clade 1 on the *B. cereus* group phylogenetic tree (Figure 1). Interestingly, Cry‐positive isolates had a significantly lower core genome allelic diversity than Cry‐negative isolates (Figure 3a,b). This was also observed when the allelic diversity of each of the 2,274 core genes of Cry‐negative isolates was plotted against the allelic diversity of the same gene in Cry‐positive isolates (Figure 3c). Only 225 of 2,274 (9.9%) core genes had a higher allelic diversity in Cry‐positive isolates, which was visualized by the circles below the proportionality line in Figure 3c. When we repeated this analysis at the clade‐level, clade 2 (with the highest prevalence of genomes harbouring predicted insecticidal toxins) also had reduced allelic diversity with respect to clades 1 and 3 (Figure 3d,e). While this approach is sample‐dependent, the difference between Cry‐positive and Cry‐negative isolates in terms of diversity cannot be explained by the clonal frame as the isolates cluster together on the tree. The contrast between high diversity in predicted insecticidal virulence factor families (Table S1) and MGEs (as inferred by Figure 2a) and lower diversity within the core genome of Cry‐positive isolates is most likely explained by lateral transfer of these elements (Figure 3).

### 3.5 Detection of plasmid genes in *B. cereus* group isolates

**Figure 4:**
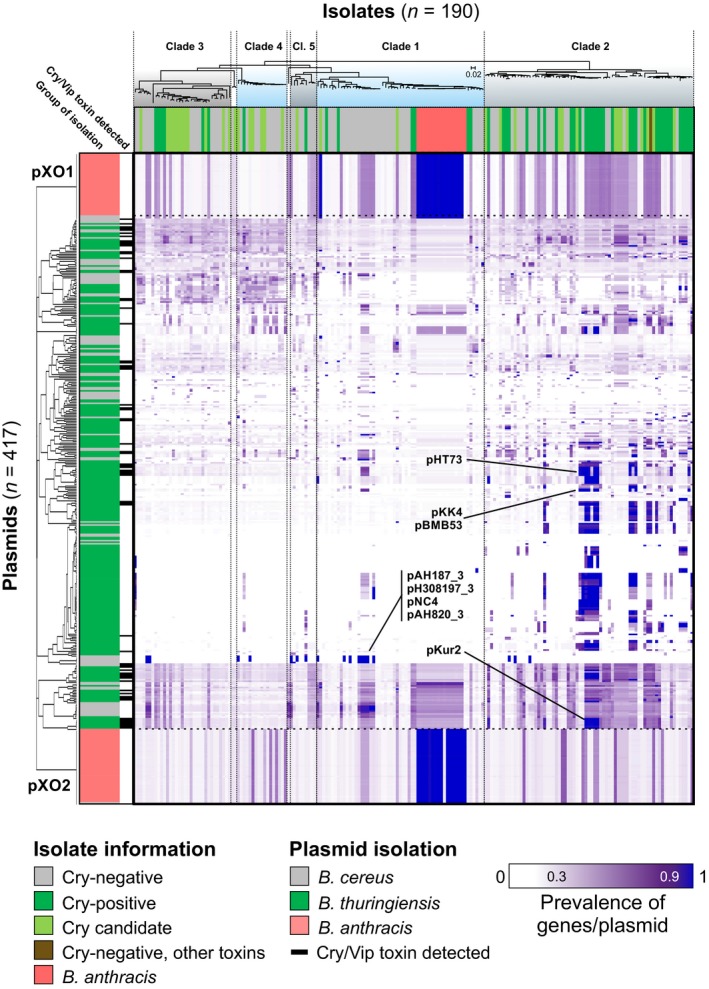
Prevalence of 410 plasmids in 190 *Bacillus cereus* group isolates. The presence of 44,759 plasmid genes from 410 plasmid reference sequences (rows) was examined in 190 genomes (columns), and the proportion of detected plasmid genes per plasmid reference sequence was calculated for each isolate. On the heatmap, blue indicates 100% of genes from that plasmid are in the genome with progressively lighter shades of purple indicating decreasing prevalence to white (fewer than 30% of genes are detected). The source of plasmid isolations (coloured row headers) and the “species” of the bacterial genome examined (coloured column headers) are given for *Bacillus anthracis* (pink), *Bacillus thuringiensis* or Cry‐positive isolate (green), *B. cereus* or Cry‐negative isolate (grey). Isolates are ordered by the tree (Figure 1a), and plasmids are clustered based on gene prevalence patterns inferred by WebGimm (Joshi et al., 2011) using the context‐specific infinite mixture model (Freudenberg et al., 2010). Names on the figure indicate known plasmid names of interest [Colour figure can be viewed at http://www.wileyonlinelibrary.com/]

**Figure 5:**
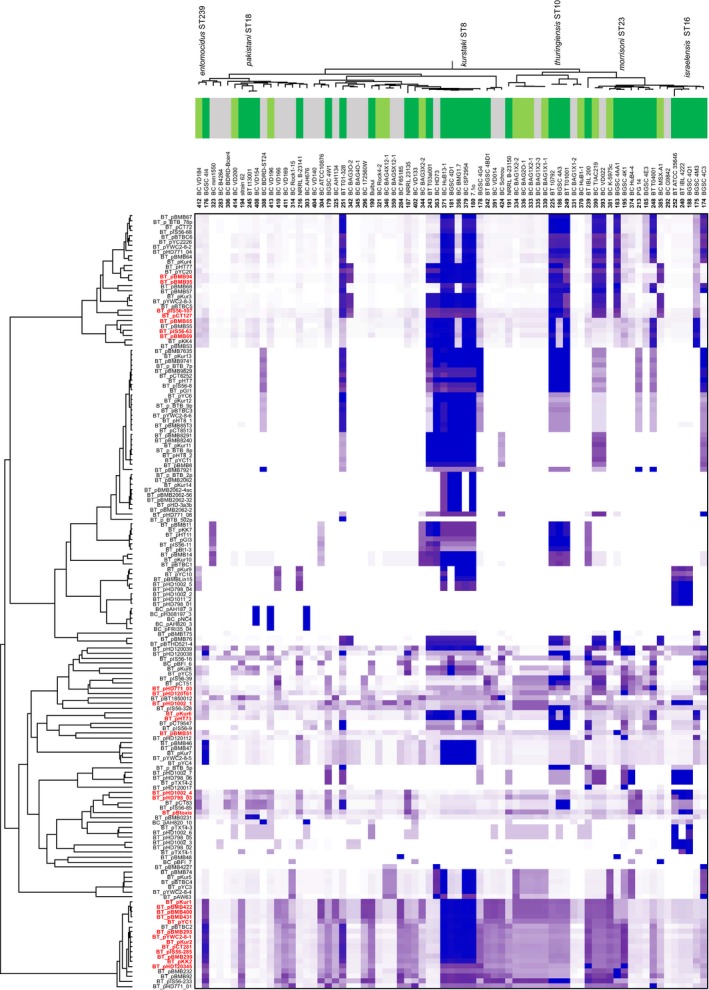
Prevalence of 116 selected plasmids in 71 clade 2 *Bacillus cereus* group isolates. Visualization is complementary to, and focuses on, specific plasmids and isolates from Figure 4. Isolates are ordered by the phylogeny from Figure 1 and plasmids from which >90% of genes were detected in at least 1 clade 2 isolate (*n* = 116) are clustered based on gene prevalence patterns inferred by WebGimm (Joshi et al., 2011) using the context‐specific infinite mixture model (Freudenberg et al., 2010). The plasmid names in red indicate Cry‐harbouring plasmids as inferred from a BtToxin_scanner analysis presented in Table S3 [Colour figure can be viewed at http://www.wileyonlinelibrary.com/]

The previous analysis, consistent with the literature on *Bt* toxins (Gonzalez et al., 1982; Mahillon, Rezsöhazy, Hallet, & Delcour, 1994), suggests mobility of virulence determinants among Cry‐habouring *B. cereus* group isolates, via plasmids or transposons. The presence of each of 48,768 genes from a reference plasmid list was examined in the 190 genomes of our data set, and the result summarized in a heatmap (Figure 4) and a table (Table 1). The total complement of genes corresponding to a particular plasmid was detected in at least one isolate genome for 53% (220/410) of reference plasmids. These included pXO1 and pXO2, but also some plasmids identified in *Bt*. Our plasmid detection was consistent with previous reports of atypical strains. One *B. cereus* isolate (strain G9241) was observed to carry a full *Ba* plasmid pXO1 and has been described before (Wilson et al., 2011). Additionally, a *Ba* strain (CDC684) was found to be missing pXO2 and has been described in the literature as having attenuated in virulence (Okinaka et al., 2011), while another one (strain A1055), missing pXO1, has been reported as atypical (Antonation et al., 2016). For 37.6% (154/410) of plasmids, between 0% and 90% of reference genes were detected in at least one isolate and only two of them (pCTC and pMC8, originally purified from *Bt* isolates) had no genes present in our data set. At least one gene from 119 plasmids was found in all 190 genomes used in this study. These included variants of the pXO2 *Ba* virulence plasmid, implying that genes from this plasmid are present in the genome of the species, either as a result of (i) homology with chromosomal core genes, or chromosomally integrated plasmid genes (Zheng et al., 2015); or (ii) homology with a widespread “plasmid core genome.” While more reference plasmid sequences are necessary to describe the full diversity within our data set, our results are consistent with the wide distribution of plasmids in the *B. cereus* group, potentially with every genome containing plasmid genes. Additionally, there were a large number of plasmids initially attributed to *Bt* that were detected in clade 2 Cry‐positive isolates (Figure 5, Table 1). Interestingly, this did not seem to be the case for Cry‐positive isolates from clades 1, 3, 4 and 5, suggesting possible clade‐specific virulence patterns.

In this analysis, we considered that the 53 closed genomes included in our data set would harbour fewer plasmid genes than draft genomes. As closing genomes require the experimental validation of the order of contigs using methods such as PCR, plasmid genes not integrated in the chromosome would be present in another amplicon at the time of DNA isolation and would be excluded from the finished closed sequence. In contrast, draft genomes are produced from the total genomic DNA of bacteria, without discrimination of plasmid or chromosomal origin. To assess the extent of missing information in genomes included in our analysis, we created a gene list including all unique genes from the 410 annotated plasmid genomes used above. A total of 7,248 genes were identified, and their presence was recorded in 53 closed genomes (Table S1) and 136 draft genomes, including 23 sequenced as part of this study (Figure S2). The 23 genomes generated in this study contained significantly more putative plasmid genes than the 53 previously published closed genomes (Figure S2, Mann–Whitney test, *p* < .0001) and 113 previously published draft genomes (Figure S2, Mann–Whitney test, *p* = .0001) which suggests that our sampling captured a large proportion of plasmid‐harbouring isolates. Interestingly, around 400 plasmid genes from our list were detected in the closed genomes (Figure S2), consistent with frequent chromosomal integration of plasmids or movement of mobile elements between plasmids and chromosomes within the *B. cereus* group.

Validation analysis was carried out on paired chromosomal and plasmid sequence from isolates where the plasmids had been purified and sequenced separately. A total of 10 plasmids were extracted from isolates present in the genomic data set of this study (Figure S3). Distinct plasmid sequences were not obtained from all isolates. This can be explained by multiple factors, including plasmid chromosomal integration or technical difficulties when isolating very large plasmids from bacteria using methods designed principally for high‐copy small plasmids. Indeed, despite methods available (Kado & Liu, 1981), obtaining correctly closed genomes of large plasmids remains a methodological challenge (Smalla, Jechalke, & Top, 2015). Nevertheless, our approach allowed plasmid and chromosomal sequence to be discriminated. We observed that seven of 10 plasmids were only detected in a single isolate, four of which from the isolate they were extracted from (Figure S3). This reflects strain‐specific plasmid acquisition. Two plasmids (pBt407 and pStrain62) were detected in additional single isolates, reflecting the possible, but limited, spread of these plasmids in the *B. cereus* group. One plasmid (pBGSC 4J4) was not detected in any isolate, reflecting the absence of the corresponding isolate in our genome data set. Notably, three plasmids from closely related isolates (p71o, pBGSC 4D4 and pBGSC 4D1) were detected in more than one isolate, all from the *kurstaki* ST8 group of clade 2 *Bt* isolates (Figure S3). This could reflect an increased spread of these plasmids and related plasmids in this ecological group, consistent with the above observations on a larger plasmid data set.

## 4 DISCUSSION

Isolate genomes within the *Bacillus cereus* group show evidence of HGT, consistent with previous work (Didelot, Barker, Falush, & Priest, 2009; Van der Auwera, Timmery, Hoton, & Mahillon, 2007; Vilas‐Bôas, Vilas‐Boas, Lereclus, & Arantes, 2008). Using the current phenotypic definition, *Bt* is recognized as being polyphyletic and as the multiple clades containing *Bt* are comprised of both *Bt* and *Bc*,* Bt* is also paraphyletic (Cardazzo et al., 2008; Didelot et al., 2009; Priest, Barker, Baillie, Holmes, & Maiden, 2004; Raymond & Bonsall, 2013; Raymond, Wyres, et al., 2010; Tourasse et al., 2011). Unsurprisingly, there are disagreements about the distinctiveness of *Bc* and *Bt,* which are compounded by the practice of applying “*B. cereus*” as a catch‐all species term when other species‐specific taxonomic data are missing. Solutions to these taxonomic inconsistencies have been debated. One view is that the entire *B. cereus* group containing *Bt*,* Bc*,* Ba*,* B. mycoides*,* B. weihenstephanensis* should be treated as one species (Helgason et al., 2000; Tourasse, Helgason, Økstad, Hegna, & Kolstø, 2006). Our genomic analysis highlights the inconsistency of *Bc*,* Ba* or *Bt* as species designations based upon phenotype comparisons, particularly for *Bc* and *Bt* that can share aspects of their ecology and do not represent discrete cohesive lineage clusters. However, all subsequent phylogenies of *B. cereus* group isolates, including this work and previous MLST studies, have shown that there are several cohesive genetically distinct clades in the *B. cereus* group (Cardazzo et al., 2008; Didelot et al., 2009; Guinebretière et al., 2008; Priest et al., 2004; Raymond, Wyres, et al., 2010; Sorokin et al., 2006; Vassileva et al., 2006; Vilas‐Boas, Sanchis, Lereclus, Lemos, & Bourguet, 2002; Zheng et al., 2017). The three major clades originally defined by MLST (*Ba* and relatives—clade 1, *B. kurstaki* and *Bc*—clade 2 and *B. weihenstephanensis*—clade 3) were recovered in this study, although the distribution of predicted insecticidal genes and of isolates identified as *B. weihenstephanensis* and *B. mycoides*, indicates that there can be additional significant heterogeneity within these clades (Figures 1 and S1).

In addition, there is abundant evidence for substantial ecological differentiation between clades, either in terms of their ability to colonize plants (Raymond, Wyres, et al., 2010; Vidal‐Quist, Rogers, Mahenthiralingam, & Berry, 2013); their carriage of virulence factors such as enterotoxins (Cardazzo et al., 2008); the risks they pose to vertebrates (Cardazzo et al., 2008; Guinebretière et al., 2010; Raymond & Bonsall, 2013) or their metabolic and growth characteristics (Guinebretière et al., 2008). Moreover, analyses of the patterns of HGT indicate that most recombination occurs within, rather than between clades, making these groups something akin to “biological species” (Didelot et al., 2009). The analysis of the distribution of *cry* genes in this study also suggests real biological differences. Clade 2 is unique in terms of both the high proportion of genomes carrying predicted insecticidal or nematicidal *cry* genes, the large number of insecticidal toxins (Cry and Vip) encoded in each genome, and the presence of a substantial number of isolates with complements of genes conferring virulence to Lepidoptera and Diptera species.

While acquisition of Cry toxin genes enables bacteria to be pathogenic to invertebrates, it imposes considerable metabolic costs on the cell both in terms of growth rate in vivo (Raymond, Davis, & Bonsall, 2007; Raymond et al., 2012) and the ability to grow or persist in soil (West, Burges, Dixon, & Wyborn, 1985; Yara et al., 1997). This high metabolic burden could explain why specialized insecticidal *cry* gene complements are largely restricted to a subset of lineages within clade 2. Reduced allelic diversity in Cry‐positive lineages could be driven by directional selection on specialized invertebrate pathogen genotypes or the clonal expansion of successful genotypes. The high cost of Cry toxin production and specialization to invertebrate hosts could explain the excellent safety record of *Bt*‐based biopesticides. Despite their close phylogenetic relationship to *Bc* isolates capable of causing diarrhoea (Raymond & Federici, 2017; Raymond, Johnston, et al., 2010; Raymond, Wyres, et al., 2010), growth in the vertebrate gut and vegetative production of enterotoxins are required for diarrhoeal food poisoning (Ceuppens et al., 2012), and production of Cry toxins is likely to hamper vegetative outgrowth considerably.

Bacterial ecology is clearly related to carriage of specific *cry* genes but a species definition based on virulence genes, rather than phenotype, offers few advantages. This is partly due to the uncertainties of gene expression but also because of the surprisingly widespread distribution of *cry* genes with no known host affiliation. For example, the parasporins *cry*31Aa, 41Aa, 42Aa 46Aa, 64A, 65A, 66A, which are cytotoxic to a range of cancer cells, were found in 5% of the isolates in this study despite having no known function in infection (Hayakawa et al., 2007; van Frankenhuyzen, 2009, 2013; Yamashita, 2005). In contrast, the *cry* toxin gene complements of genomes in clade 2 typically have readily identifiable host ranges comprising a particular insect order or nematodes (Figure 1b), again suggesting that isolates in this clade in particular are well adapted to exploiting invertebrate hosts (Raymond & Bonsall, 2013; Raymond, Johnston, et al., 2010; Raymond, Wyres, et al., 2010). Arguably, any revision of the nomenclature would be most informative if it could reflect both phylogenetic affiliation and presence of Cry toxin inclusions.

Our analysis of plasmid distribution across the group revealed important patterns, illustrating the relationship between key plasmids and the genomes of specialized pathogens. Substantial sharing of near complete plasmids across genomes (Figure 4, Table 1) can indicate clonal expansions, sampling/sequencing bias of particular genotypes or horizontal transfer of plasmids between distinct lineages. The clonal expansion of *Ba* ST1 is well established (Keim et al., 2009; Zwick et al., 2012); however, the clonal expansion of the invertebrate pathogen *Bt subsp. kurstaki* (ST8), indicated by the central block of high plasmid sequence homology in clade 2 in Figure 4, is less well appreciated. This is the most frequently recorded genotype in the pubmlst database (Jolley, Chan, & Maiden, 2004). It is also the most common genotype/serotype found on plants in a number of countries (Damgaard, Hansen, Pedersen, & Eilenberg, 1997; Maduell, Callejas, Cabrera, Armengol, & Orduz, 2002; Ohba, 1996; Raymond, Wyres, et al., 2010), possibly due to its ability to colonize plants from the soil (Raymond, Wyres, et al., 2010). Therefore, in terms of global abundance, the clonal expansion of *Bt. subsp. kurstaki* ST8 dwarfs that of *Ba*. The other abundant clone in our genomic data set corresponds to ST26, or the “emetic cluster” of cereulide‐producing *Bc* that is capable of causing lethal food poisoning (Priest et al., 2004; Vassileva et al., 2007). In this case, the strong representation of this cluster in the genomic database may be due to sampling bias.

If plasmid–bacteria associations are driven by co‐evolution, we predicted that particular plasmids should be associated with particular lineages. This was true for some plasmids (Figure 4, Table 1). The pXO2 plasmid of *Ba* was phylogenetically restricted to *Ba*, although plasmids with homology to pXO1 are widely distributed in clades 1 and 2 (Hu, Swiecicka, et al., 2009; Zheng et al., 2013). A large number of plasmids, including the Cry‐bearing plasmids which possess *orf156/157* minireplicons (Zheng et al., 2013), were phylogenetically restricted to clade 2 (Figures 4 and 5), as has been found previously (Zheng et al., 2017). Infectious cooperation, on the other hand, predicts that conjugative plasmids carrying social genes such as Cry toxins should be widely distributed across clades and show evidence of recent horizontal transfer. Several groups of plasmids that were widely distributed either within or between clades and which had conserved gene content, were observed. However, several of these are small putatively parasitic plasmids such as the mobilizable 3 kb plasmid sequenced from strains present in the ST26 emetic cluster (synonymous with pNC4), note that this plasmid does not carry the cereulide toxin (Hattori, Yamashita, Toh, Oshima, & Shiba, 2012) (Figure 4). Within clade 2, the widely distributed mobile elements with the highest levels of conserved gene content are 60–80 kb transposase‐rich plasmids related to pKur6 and a class of ≈8 kb plasmids related to pKur 11, 12 and 13 (Figure 5). These are shared widely among *Bt* subspecies and isolates infectious for Lepidoptera and Coleoptera (*kurstaki* ST8*, thuringiensis* ST10*; morrisoni* ST23 *darmastadiensis* BGSC 4M3*; alesti 4C3,* T01‐328, T0‐40001) (Figure 5). These plasmids are not associated with Cry toxin genes, and their association with particular hosts could simply be the result of the increased opportunities for plasmid transfer between strains that share an ecological niche in insect cadavers (Vilas‐Bôas et al., 2008).

For plasmids associated with the production of Cry toxins, we also see that distantly related lineages within clade 2 can share closely related plasmids, indicating recent horizontal transfer. Plasmids closely related to pBtoxis, which carries multiple mosquitocidal Cry proteins and was originally described in *Bt. israelensis* 4Q1 (Berry et al., 2002), are found in *Bt. morrisoni* PG14 and nematicidal *Bt. pakistani* ST17. A group of plasmids related to the *kurstaki* Cry toxin 300 kb mega‐plasmid pKur2 are very widely distributed among nearly all other *Bt* within clade 2. The 85 kb plasmids carrying single Cry1A toxins (pHT73 and pKur6) with *ori44* minireplicons are also shared by several distinct lineages (*kurstaki* ST8, *thuringiensis* ST10*; darmastadiensis* BGSC 4M3), plasmids with these minireplicons have been found widely across the *B. cereus* group. Not only are Cry toxin plasmids present in distinct lineages but sister taxa, for example *kurstaki* HD73 and HD1; *buibui* and BcRock42; *entomocidus* BSSC 4I4 and BcVD184, may or may not carry mega‐plasmids, a pattern also indicating recent loss or acquisition. This pattern of recent transfer is consistent with infectious cooperation of Cry toxins, which are known public goods (Raymond et al., 2012). Nevertheless, gene content in these large plasmids is very unstable, indicating that costly social genes may be quickly lost in many lineages, perhaps in those not fully adapted to a specialized pathogenic niche.

Together, our analyses describe multiple groups of specialized pathogens (*Ba* and several *Bt* lineages) that are associated with phylogenetically restricted virulence plasmids. This stratification among mobile plasmids, and the conserved allelic content, suggests that particular plasmid‐chromosome combinations result in clonal expansion of successful pathogens. The distribution of virulence plasmids in particular suggests an association that emerges out of the ability of plasmids to rapidly change gene content and associate with new chromosomes (Keim & Wagner, 2009) and the subsequent proliferation of successful plasmid‐chromosome combinations. While plasmid/bacteria co‐evolution may not appear consistent with regular transfer of plasmids during infectious cooperation, we do in fact see evidence for repeated transfer and loss of plasmids carrying cooperative Cry genes at a different taxonomic scale, namely within clade 2 (Rankin et al., 2010; Raymond et al., 2012). More widespread evidence of recent horizontal transfer may not be present, either because these plasmids are restricted to one clade or because of lack of ecological opportunities for transfer in lines that are more distantly related. Infectious cooperation, of course, may occur at the level of MGEs (integrons, transposons) within plasmids or result in chromosome/plasmid combinations that are highly unstable due to genetic conflict. The most striking finding from the plasmid distribution data set was the very rapid and dynamic change in plasmid gene content between closely related genomes. Coupled with the extremely open pan‐genomic structure seen in this study and the evidence of widespread exchange of genes between plasmid and chromosome in previous work (Zheng et al., 2015), this level of variability suggests that plasmids could be gaining and shedding genes on ecological timescales—a process that could explain hitch‐hiking to high frequencies (Bergstrom et al., 2000) as well as a means of rapidly responding to selective bottlenecks imposed by host colonization.

## CONFLICT OF INTEREST

Authors declare no conflict of interest.

## AUTHOR CONTRIBUTION

G.M., B.R. and S.K.S. wrote paper/designed study; G.M., L.M., D.J.W. and E.M. performed analyses; B.R. sampling and DNA extraction; B.P., S.L., R.B. and K.A.J. DNA sequencing, assembly and archiving. B.R. Designed study; Extracted DNA; Wrote paper.

## DATA ACCESSIBILITY

Raw reads and assembled contiguous sequences of bacterial and plasmid genomes generated in this study are accessible and associated with NCBI BioProject PRJNA395643.
