# Supplementary figures and images for: Lineage‐specific plasmid acquisition and the evolution of specialized pathogens in Bacillus thuringiensis and the Bacillus cereus group
Source: Mol Ecol. 2018 Apr 2;27(7):1524–40. doi: 10.1111/mec.14546 (PMC5947300; doi:10.1111/mec.14546)

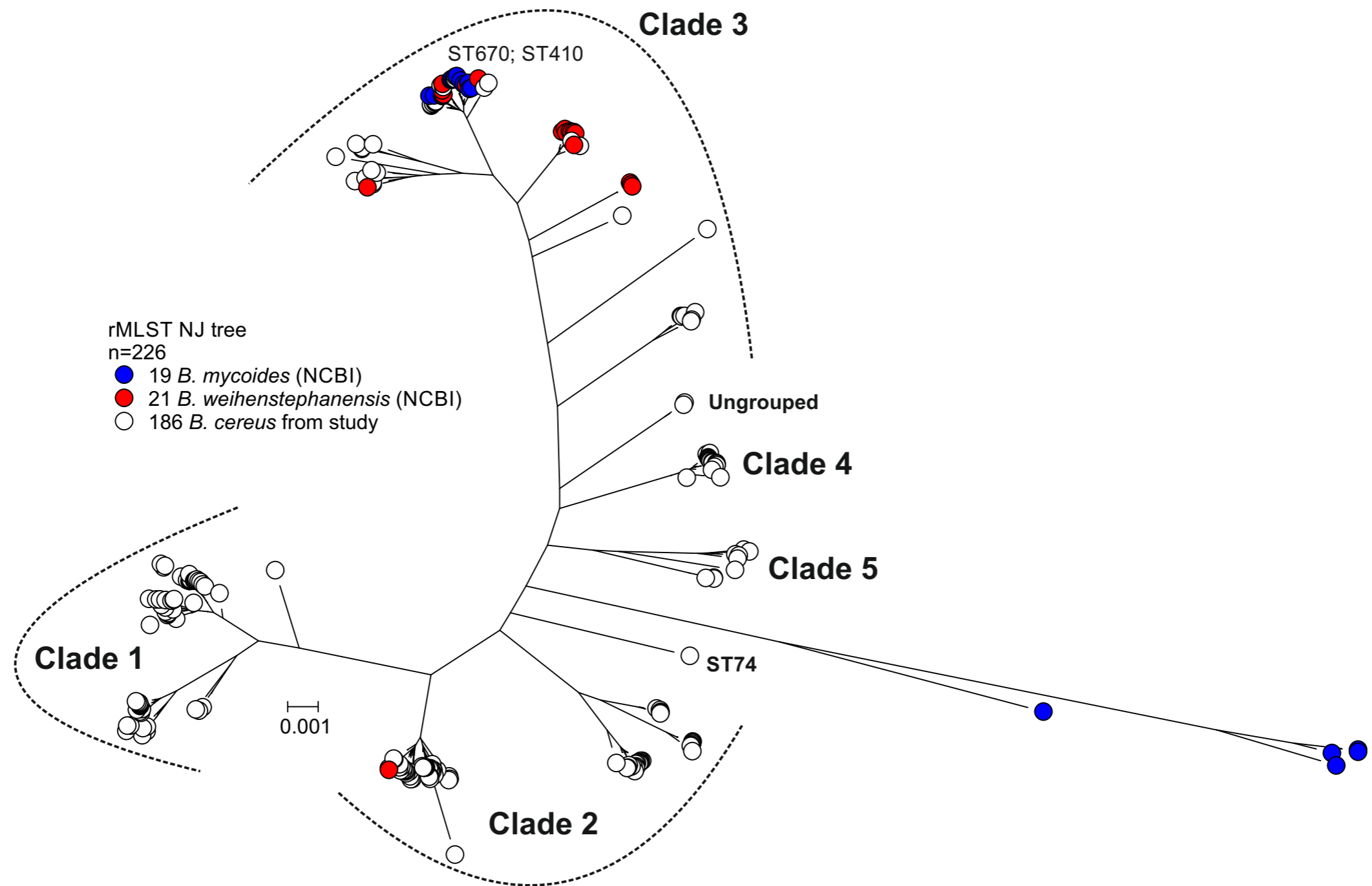

Supplement: Supplementary file 1 [file MEC-27-1524-s001.pdf]

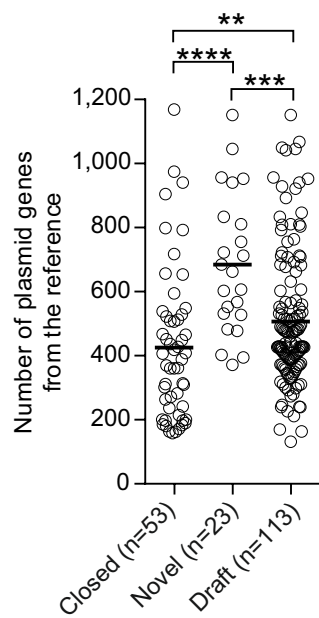

Supplement: Supplementary file 2 [file MEC-27-1524-s002.pdf]

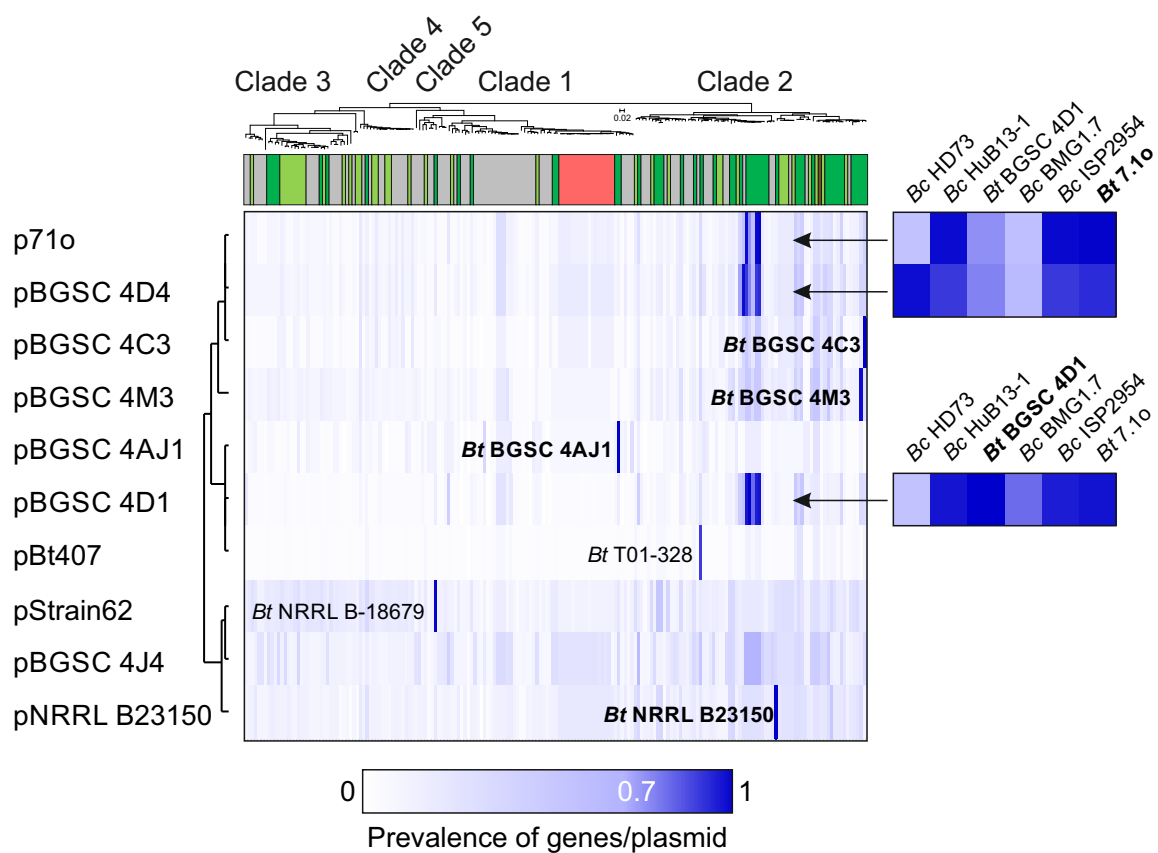

Supplement: Supplementary file 3 [file MEC-27-1524-s003.pdf]

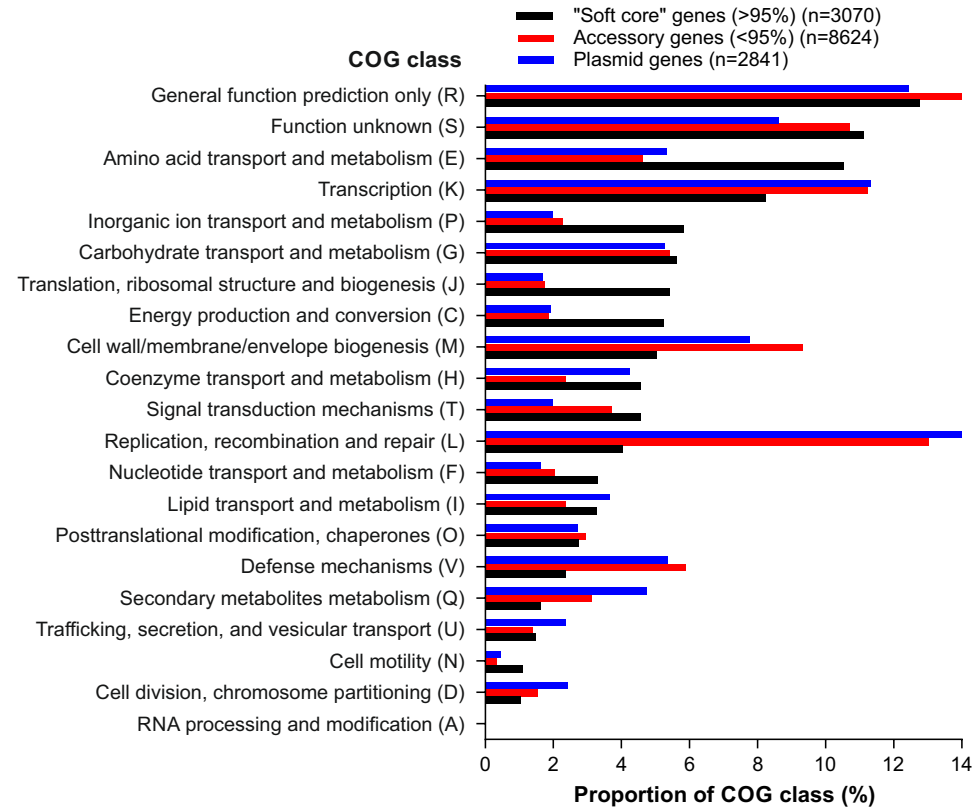

Supplement: Supplementary file 4 [file MEC-27-1524-s004.pdf]
